# Terahertz stimulated parametric downconversion of a magnon mode in an antiferromagnet

**DOI:** 10.1126/sciadv.adv3757

**Published:** 2025-05-21

**Authors:** Zhuquan Zhang, Yu-Che Chien, Man Tou Wong, Frank Y. Gao, Zi-Jie Liu, Xiaoxuan Ma, Shixun Cao, Edoardo Baldini, Keith A. Nelson

**Affiliations:** ^1^ Department of Chemistry, Massachusetts Institute of Technology, Cambridge, MA 02139, USA.; ^2^ Department of Physics, The University of Texas at Austin, Austin, TX 78712, USA.; ^3^ Department of Physics, Materials Genome Institute and International Center for Quantum and Molecular Structures, Shanghai University, Shanghai 200444, China.

## Abstract

Parametric amplification, where one signal is enhanced by the action of another, offers both practical utility for boosting weak signals and fundamental insights into the nonlinear coupling between degrees of freedom. In condensed matter systems, interactions between collective modes offer avenues for nonlinear coherent manipulation of coupled excitations and quantum phases. Antiferromagnets, with inherently coupled magnon modes, provide a promising platform for nonlinear control of spin waves and magnetization. However, nonlinear magnon-magnon interactions have been only partially elaborated, leaving key gaps in the prospects for potential ultrahigh-bandwidth magnonic signal processing. Here, we excite two distinct coherent magnon modes in an antiferromagnet and find that the magnon mode with a lower frequency undergoes amplification when the higher-frequency mode is driven. We unveil the nonlinear excitation pathways of this stimulated parametric downconversion process by using polarization-selective two-dimensional terahertz spectroscopy. Our work provides fundamental insights into nonlinear magnonics in antiferromagnets, laying the groundwork for forthcoming spintronic and magnonic devices.

## Teaser

Two-dimensional terahertz spectroscopy unveils stimulated parametric downconversion of a coherent magnon.

## INTRODUCTION

**Fig. 1.**
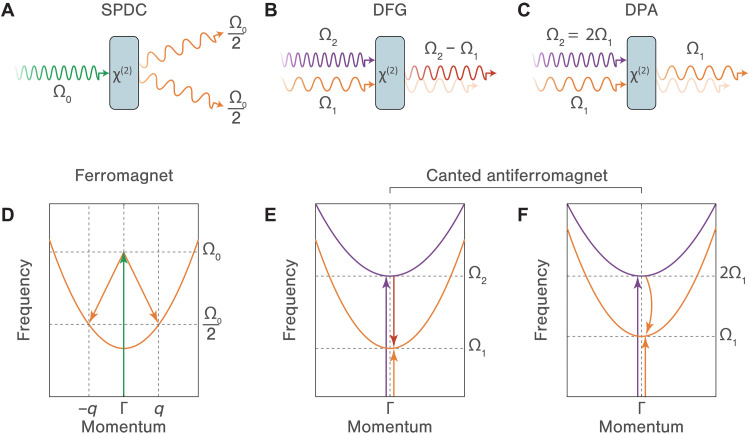
Nonlinear optics and nonlinear magnonics. (**A**) Spontaneous parametric downconversion (SPDC). In the degenerate limit, a pump wave with frequency $\Omega_{0}$ generates a pair of optical photons at half of the pump frequency (i.e., $\frac{\Omega_{0}}{2}$), conserving both momentum and energy. (**B**) Difference frequency generation (DFG). A signal wave with frequency $\Omega_{1}$ and a pump wave with frequency $\Omega_{2}$ generate an idler wave at frequency $\Omega_{2}-\Omega_{1}$. (**C**) Degenerate parametric amplification (DPA). A signal wave with frequency $\Omega_{1}$ and a pump wave with frequency $\Omega_{2}=2\Omega_{1}$ results in the amplification of the signal wave. (**D**) Parametric excitation of magnon modes in a ferromagnet. A strong microwave field with frequency $\Omega_{0}$ directly couples to two counterpropagating spin waves at wave vectors ±q with half of the pump frequency $\frac{\Omega_{0}}{2}$. (**E**) DFG of two distinct magnon modes in a canted antiferromagnet. Coherent excitations of both magnon modes lead to photon emission at the frequency $\Omega_{2}-\Omega_{1}$. (**F**) DPA of a coherent magnon mode in a canted antiferromagnet. When $\Omega_{2}=2\Omega_{1}$, driving both magnon modes leads to the amplification of the lower-frequency magnon.

Parametric excitation and amplification are ubiquitous phenomena in nonlinear systems, occurring when a specific parameter is varied to drive or amplify a coupled degree of freedom. One salient manifestation of this principle is the amplification of light through parametric nonlinear optical processes (*1*). If a second-order nonlinear optical medium is pumped by a light wave of frequency $\Omega_{0}$, spontaneous parametric downconversion (*2*, *3*) can be initiated to generate an entangled photon pair, for instance, at half the pump frequency (i.e., $\frac{\Omega_{0}}{2}$), as shown in Fig. 1A. On the other hand, if a signal wave with frequency $\Omega_{1}$ is introduced alongside the pump wave at frequency $\Omega_{2}$, the stimulated version of the downconversion process leads to difference frequency generation (DFG) of an idler wave at frequency $\Omega_{2}-\Omega_{1}$ (see Fig. 1B). As a result, a special condition arises when the signal and idler waves are indistinguishable and the pump frequency is precisely double the signal frequency (i.e., $\Omega_{2}=2\Omega_{1}$). Under these circumstances, as illustrated in Fig. 1C, the nonlinear interaction amplifies the signal wave alongside a degenerate idler wave at the same frequency, an effect referred to as degenerate parametric amplification (*4*). Such a nonlinear process is a stimulated counterpart of spontaneous parametric downconversion and has been extensively harnessed to attain desired optical functionalities in fields ranging from optical communication (*5*–*7*) and quantum optics (*8*–*10*) to quantum information processing (*11*, *12*).

In magnetically ordered systems, the generation, manipulation, and detection of spin waves, as well as their quanta (magnons), hold paramount promise for emerging information and signal processing platforms based on spintronics and magnonics (*13*). Within this framework, controlling nonlinear interactions among magnon modes, a paradigm known as nonlinear magnonics (*14*), enables the creation of magnonic states beyond what can be achieved in the linear excitation regime (*15*–*17*). This concept bears close analogies to nonlinear optics. One manifestation of this parallel is the parametric excitation of magnons in ferromagnets (*18*, *19*). Such a nonlinear process involves interactions between a microwave photon and two magnons from a single spin-wave branch but at varying wave vectors (Fig. 1D). In contrast, the presence of high-frequency magnon modes in complex antiferromagnets (*20*–*23*) appears to be a highly desirable yet challenging candidate for the nonlinear coherent control of magnon-magnon interactions at terahertz (THz) frequencies (*24*, *25*). Only recently has it been realized that THz excitations of two individual magnon modes in a canted antiferromagnet can lead to coherent photon emission at the difference frequency (*16*), as illustrated in Fig. 1E. Such a nonlinear interaction can amplify the lower-frequency magnon coherence if the parametric resonance condition is satisfied (see Fig. 1F). This mechanism differs from previously established examples involving magnon harmonics (*23*, *26*, *27*), conversion (*15*, *17*), and coupling to phonons (*28*), which all focus on driving nonlinear responses absent in the linear regime. In contrast, the stimulated version of parametric amplification offers a route to selectively enhance weak magnonic signals, making it highly desirable for both fundamental studies and practical applications.

Here, we devise a protocol to investigate the stimulated parametric downconversion of a coherent magnon mode using polarization-selective two-dimensional (2D) THz spectroscopy. This technique allows us to disentangle the nonlinear responses of various origins and provides sensitivity to the polarization of the desired nonlinear signal. Following this approach, we unveil two distinct nonlinear channels for the amplification of a coherent magnon mode in a canted antiferromagnet and discover a crossover from magnonic DFG to degenerate parametric amplification, a unique pathway to achieve tailored magnonic responses enabled by magnon-magnon interactions.

## RESULTS

As a test bed, we select the antiferromagnetic insulator ErFeO_3_, which crystallizes in an orthorhombic perovskite structure with space group *Pbnm*. Below the Néel transition temperature of approximately 643 K and above the spin reorientation transition temperature of 96 K (*29*), ErFeO_3_ orders in the $\Gamma_{4}$ magnetic phase (*25*). In this phase, the spins of neighboring Fe ions are antiferromagnetically aligned along the *a* axis, but slightly canted toward the *c* axis, giving rise to a weak net magnetization **M** along the *c* axis. Consequently, two distinct magnon modes exist in the THz frequency range, each following specific selection rules. In the linear response regime, the quasi-antiferromagnetic (qAFM) mode, which corresponds to the oscillation of the magnetization amplitude, is driven by THz radiation with $H_{\text{THz}}∥c$, while the quasi-ferromagnetic (qFM) mode, which corresponds to the precession of the net magnetization, is excited by THz radiation with $H_{\text{THz}}⊥c\;$ (*29*, *30*). Although more complex selection rules govern nonlinear responses driven by the THz electric or magnetic components, these responses are substantially weaker than the dominant linear responses and are not observed in our experiment, as will be shown later. Notably, the spin canting in ErFeO_3_ breaks the time-reversal symmetry, thereby allowing second-order nonlinear responses based on magnon-magnon interactions. Moreover, the magnon frequencies are broadly tunable by changing the temperature in the $\Gamma_{4}$ phase, making ErFeO_3_ a promising candidate for observing magnonic parametric amplification.

**Fig. 2.**
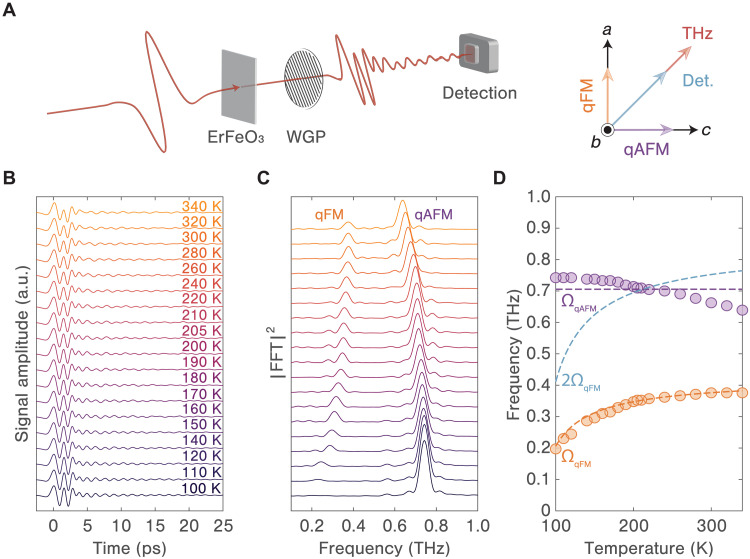
Temperature-dependent linear magnon response. (**A**) Sketch of the experimental setup for time-domain THz spectroscopy measurements. The magnetic field orientation of the THz pulses and the WGP are set at a 45° angle relative to both *a* and *c* axes. (**B**) Time domain THz signals showing the excitation of both magnon modes across a range of temperatures. The initial double-peak profile in the primary THz signals arises from a temporal walk-off of the THz field components along different crystallographic axes. (**C**) Fourier transforms of the oscillatory responses following the primary THz peaks presented in (B). (**D**) Experimentally derived frequencies for both the qFM (orange dots) and qAFM (purple dots) magnon modes plotted against temperature. Fits are based on the uniform two-spin model, represented by dashed lines in corresponding colors. The dashed blue line signifies double the frequency of the qFM mode, which intersects with the qAFM mode frequency at approximately 200 K.

As an initial step, we elucidate the magnon signal by performing time-domain THz spectroscopy measurements on a (010)-cut ErFeO_3_ crystal at varying temperatures. As depicted in Fig. 2A, the THz pulse is linearly polarized at 45° relative to both the *a* and *c* axes. A wire-grid polarizer (WGP) is placed after the sample to detect only the polarization of the emitted signal parallel to that of the incident THz pulse. Under these experimental conditions, both qAFM and qFM modes are excited and detected, manifesting in the time domain as two overlapping oscillatory responses following initial peaks from the incident THz field (see Fig. 2B). Fourier transformations of the time domain signals shown in Fig. 2C reveal the evolution of the magnon frequencies over a broad temperature range. As the sample temperature is reduced from 340 to 100 K, the qFM mode undergoes notable softening, especially as it approaches the spin reorientation transition temperature. Conversely, the qAFM mode only exhibits a slight hardening as the temperature decreases, mainly due to moving farther from the Néel transition at a considerably elevated temperature. To achieve a more comprehensive understanding of the magnon frequency shifts, we model the system with a uniform two-spin Hamiltonian

$$ℋ=\mathrm{nJ}S_{1}\cdot S_{2}+nD\cdot \left(S_{1}\times S_{2}\right)-\sum_{i=1,2}\left(K_{a}S_{\mathrm{ia}}^{2}+K_{c}S_{\mathrm{ic}}^{2}\right)$$

where the first term denotes the antiferromagnetic exchange interaction, the second term represents the Dzyaloshinskii-Moriya interaction leading to spin canting, and the third term describes the magnetocrystalline anisotropy energy. Here, $S_{1}$ and $S_{2}$ represent the two sublattice spins, n=6 is the count of neighboring spins, J and D are the symmetric exchange and antisymmetric exchange constants, respectively, while $K_{a}$ and $K_{c}$ are the magnetic anisotropy components aligned with the *a* and *c* axes, respectively. $K_{c}$ is temperature dependent to account for the spin reorientation transition. The magnon frequencies at the zone center are determined as

$$\hbar \Omega_{\text{qFM}}=2S{\left[n\left(J+K_{a}\right)\left(K_{a}-K_{c}\right)\right]}^{\frac{1}{2}}$$

and

$${\hbar \Omega }_{\text{qAFM}}=S{\left[4\mathrm{nJ}K_{a}+4K_{a}\left(K_{a}-K_{c}\right)+n^{2}D^{2}\right]}^{\frac{1}{2}}$$

These expressions are used to fit the experimentally observed magnon frequencies by adopting reported values of J and D (*29*, *31*, *32*). All parameter values are reported in table S1. The fits along with the experimental magnon frequencies are plotted as a function of temperature in Fig. 2D. Although this model does not account for the redshift of the qAFM mode frequency toward the Néel transition temperature, it aligns well with the qFM mode frequencies across all temperatures. Notably, at about 200 K, the qAFM mode frequency matches twice the qFM mode frequency, i.e., $\Omega_{\text{qAFM}}=2\Omega_{\text{qFM}}$, serving as a benchmark for the parametric resonance condition.

**Fig. 3.**
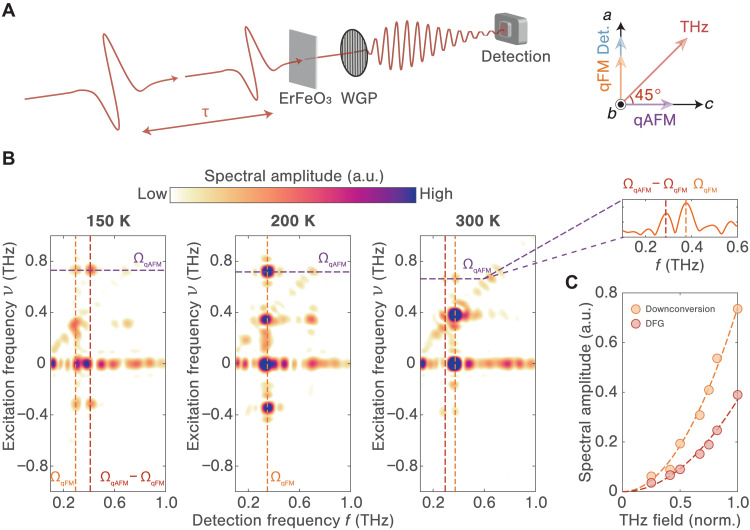
Polarization-selective 2D THz spectroscopy measurements. (**A**) Sketch of the experimental setup for the polarization-selective 2D THz spectroscopy measurements. The magnetic field orientation of both THz pulses is set at a 45° angle relative to both *a* and *c* axes. The WGP is adjusted to allow for the detection of magnetization emissions along the *a* axis, while rejecting those along the *c* axis. (**B**) Representative 2D THz spectra at 150 (left), 200 (middle), and 300 K (right). For the 300 K data, a spectral line cut along the excitation frequency $f=\Omega_{\text{qAFM}}$, is shown. Detection frequencies are indicated by the dashed red line for $f=\Omega_{\text{qAFM}}-\Omega_{\text{qFM}}$ and the dashed orange line for $f=\Omega_{\text{qFM}}$. (**C**) THz magnetic field dependencies of the spectral amplitudes for both DFG (red dots) and downconversion (orange dots) signals at 300 K, accompanied by their respective quadratic fits (dashed lines).

Having established the magnon responses in the linear response regime across a wide range of temperatures, we now examine the possibility for nonlinear parametric amplification of magnon coherences. We use a pair of nearly identical, linearly polarized, high-field THz pulses, each carrying a peak magnetic field strength of approximately 0.17 T, which interact sequentially with the sample (see note S1). The magnetic field components of these THz fields are oriented 45° relative to the *a* and *c* crystallographic axes, ensuring the excitation of both magnon modes. The WGP is adjusted to exclusively select the THz magnetic field emission along the *a* axis. As a result, only signals bearing a nonzero emission along the qFM mode axis are detectable, as depicted in Fig. 3A. By varying the inter-pulse delay time τ and recording the time-dependent signal fields induced by either or both THz pulses, leveraging a state-of-the-art single-shot detection technique (*33*, *34*), we can isolate the nonlinear signals from coherent magnon emissions, i.e., S(τ,t). A subsequent 2D Fourier transformation of these nonlinear responses allows for the identification of signals originating from distinct excitation pathways (*23*, *26*, *35*–*44*), which manifest as individual peaks in the 2D frequency-frequency correlation maps, S(ν,f).

Figure 3B shows the 2D THz spectra obtained at a few selected temperatures, namely, 150, 200, and 300 K. These temperatures correspond to conditions below, at, and above the parametric resonance, with magnon frequencies: $\Omega_{\text{qAFM}}>2\Omega_{\text{qFM}}$, $\Omega_{\text{qAFM}}=2\Omega_{\text{qFM}}$, and $\Omega_{\text{qAFM}}<2\Omega_{\text{qFM}}$, respectively. Although these spectra feature multiple peaks (see note S2 for detailed description), our analysis focuses primarily on peaks with the excitation frequency equal to the qAFM mode frequency, i.e., $\nu =\Omega_{\text{qAFM}}$. Divergence from the parametric resonance condition results in the appearance of two distinct peaks in the data. The detection frequencies of these peaks align with the qFM mode frequency, i.e., $=\Omega_{\text{qFM}}$, and with the result of DFG from the two magnon modes, i.e.,$f=\Omega_{\text{qAFM}}-\Omega_{\text{qFM}}$, respectively. The former peak is ascribed to a downconversion process wherein the first THz pulse excites the qAFM mode at frequency $\Omega_{\text{qAFM}}$, and this is succeeded by a secondary field interaction with frequency component at the difference frequency (i.e., $\Omega_{\text{qAFM}}-\Omega_{\text{qFM}}$), thus transferring the coherence from the qAFM mode to the qFM mode at frequency $\Omega_{\text{qFM}}\;$ (*44*). The latter corresponds to the previously mentioned magnonic DFG resulting from magnon-magnon interaction after the first THz pulse excites the qAFM mode and the second excites the qFM mode (*16*). Field-dependence measurements at 300 K show that the amplitudes of both peaks scale quadratically with the incident THz field strength (see Fig. 3C), and therefore both signals originate from second-order nonlinear processes. Thus, contributions from higher-order electric field–driven processes are excluded. Furthermore, these signals vanish when the WGP is rotated by 90° to select the THz magnetic field emission along the *c* axis (see fig. S3). This observation confirms that the DFG response shares the same symmetry as the qFM magnon emission and acts as a precursor to degenerate parametric amplification. At 200 K, the DFG frequency coincides with the qFM mode frequency, resulting in a single strong peak, indicative of degenerate parametric amplification.

**Fig. 4.**
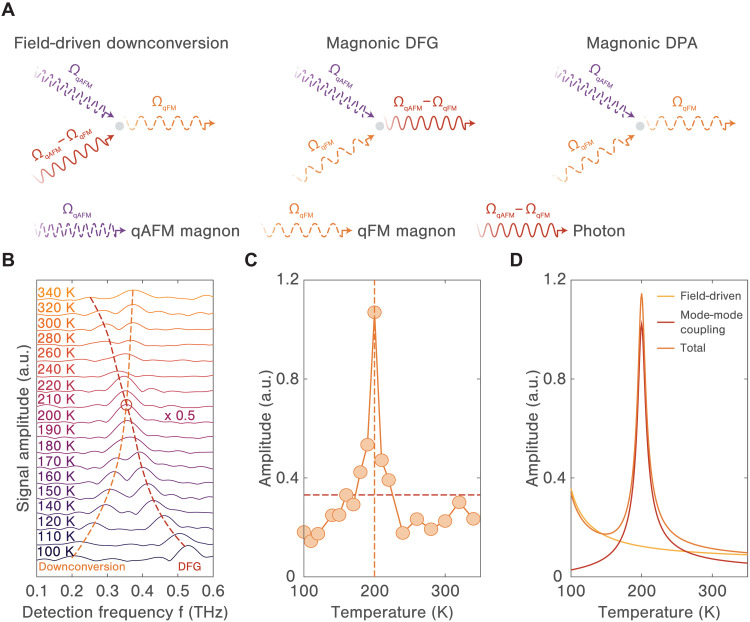
Stimulated parametric downconversion of the qFM mode. (**A**) Diagrammatic representations of field-driven downconversion (left), magnonic DFG (middle), and magnonic degenerate parametric amplification (right). The qAFM (qFM) magnon coherence is indicated by a dashed purple (orange) wavy line, while the photon field at the difference frequency (i.e., $\Omega_{\text{qAFM}}-\Omega_{\text{qFM}}$) is shown by a solid red wavy line. (**B**) Spectral line cuts along the excitation frequency $\nu =\Omega_{\text{qAFM}}$ across a broad range of temperatures. The spectral amplitude at 200 K is multiplied by 0.5 for better visualization. The dashed curves indicate the downconversion frequencies (i.e., $\Omega_{\text{qFM}}$) increasing with temperature) and the DFG frequencies (decreasing with temperature). (**C**) Temperature dependence of the amplitude of the downconversion signal. The amplitude peaks at 200 K (blue dashed line), where the qAFM mode frequency matches twice the qFM mode frequency ($\Omega_{\text{qAFM}}=2\Omega_{\text{qFM}}$). The red dashed line shows the averaged amplitude of the DFG signals at temperatures away from 200 K, which is smaller than half the peak amplitude at 200 K. (**D**) Computed downconversion amplitude contributed by the field-driven mechanism (orange) and the parametric amplification mechanism due to magnon-magnon interactions (red line). The blue line indicates the sum of the two contributions.

Three main nonlinear responses are thus identified, field-driven downconversion, magnonic DFG, and degenerate parametric amplification, all of which are second-order in the THz field but arise from distinct photon-magnon interactions (see Fig. 4A). Field-driven downconversion occurs when the combined magnon field ($\Omega_{\text{qAFM}}$) and photon field ($\Omega_{\text{qAFM}}-\Omega_{\text{qFM}}$) excite the qFM mode ($\Omega_{\text{qFM}}$) without any direct nonlinear coupling between magnon modes. In contrast, magnonic DFG process requires both the qAFM and qFM magnon modes to be excited so that their interaction produces a photon field that emits at the difference frequency ($\Omega_{\text{qAFM}}-\Omega_{\text{qFM}}$). Under the parametric resonance condition (i.e., $\Omega_{\text{qAFM}}=2\Omega_{\text{qFM}}$), the nonlinear mixing of both magnon modes directly couples to the qFM mode, leading to amplification of the magnon coherence. For a more quantitative analysis, we select a line cut across the excitation frequency of the qAFM mode, i.e., $\nu =\Omega_{\text{qAFM}}$, and represent the resulting spectra across a wide temperature range in Fig. 4B. Below 200 K, when $\Omega_{\text{qAFM}}>2\Omega_{\text{qFM}}$, the downconversion peak has a frequency lower than that of the DFG peak. Conversely, at temperatures exceeding 200 K, the frequency of the DFG peak is lower. Precisely at 200 K, which coincides with the parametric resonance condition, the two peaks converge to form a single peak with substantially enhanced spectral amplitude. These features are confirmed in Fig. 4C, which depicts the temperature-dependent amplitude evolution of the downconversion peak, i.e., [$\Omega_{\text{qFM}},\Omega_{\text{qAFM}}$]. It is important to note that the downconversion signal is not only attributed to the THz field–assisted process previously mentioned but also includes contributions from degenerate parametric amplification, occurring when the magnonic DFG resonates with the qFM mode. The data clearly show that the downconversion signal persists across all examined temperatures, peaking at 200 K, in accordance with the parametric resonance condition. Notably, this enhancement cannot be explained by the linear superposition of the downconversion and DFG signals, as the peak amplitude at 200 K far exceeds the sum of these signals at temperatures away from 200 K. This observation is corroborated by numerical solutions of the nonlinear equations of motion that describe the spin dynamics of the system, and the results are fitted with a model that integrates both the field-driven downconversion mechanism and the parametric amplification due to magnon-magnon interactions (see note S5). The simulation precisely reproduces the peak behavior as well as the general trend (see Fig. 4D). Notably, this pronounced peak indicates an enhanced magnon downconversion process, which can only be attributed to magnonic degenerate parametric amplification, a particular manifestation of magnon-magnon interactions, thus underscoring its significance to the observed phenomenon. Unlike the field-driven downconversion, degenerate parametric amplification continues even after the THz driving field ceases, as evidenced by the gradual increase in signal amplitude shown in fig. S4. Although these observations bear similarity to the previously reported magnon upconversion process, which likewise involves nonlinear interactions between two qFM modes and one qAFM mode and shows enhancement at the parametric resonance, each process follows a different nonlinear pathway. The upconversion process involves only driving the qFM mode to couple to the qAFM mode, while degenerate parametric amplification requires excitation of both the qAFM and qFM modes, analogous to its optical counterpart involving both pump and signal waves.

## DISCUSSION

Our spectroscopic measurements unveil a crossover from the magnonic DFG to the degenerate parametric amplification of two distinct magnon modes in an antiferromagnet. The parametric amplification identified in this study is second-order relative to the THz fields and becomes notable when the frequency-matching condition is satisfied. This behavior contrasts with previous observations in ferromagnets (*18*, *19*), ferrimagnets (*45*), and synthetic antiferromagnets (*46*) subjected to microwave and optical excitations, where the amplification occurs only after surpassing a specific instability threshold. As such, our method heralds a unique approach for the nonlinear coherent control of magnon modes in antiferromagnets, which are foundational to the design of magnonic oscillators and amplifiers. For instance, by using two multicycle THz pulses with orthogonal polarizations and distinct frequency components, namely, $\Omega_{\text{qAFM}}$ and $\Omega_{\text{qFM}}$, one could leverage degenerate parametric amplification to enhance efficiency and exploit magnon-magnon interactions to amplify the lower-frequency THz signal under the parametric resonance condition (i.e., $\Omega_{\text{qAFM}}=2\Omega_{\text{qFM}}$). Analogous to the degenerate parametric amplification of light, the stimulated parametric downconversion of antiferromagnetic magnons opens up avenues for generating exotic magnonic states, such as magnon squeezing (*47*) and Bose-Einstein condensation of magnons (*48*). In many antiferromagnets and altermagnets with either spontaneous or externally induced spin canting, the magnon frequencies are sensitive not only to temperature but also to other external parameters, including magnetic field (*49*, *50*), strain (*51*), and cavity engineering (*52*, *53*), making the prospects for exploitation of parametric amplification generally available, even at room temperature. More broadly, although parametric amplification of collective excitations has been proposed or observed in materials for phonons (*54*–*56*), Josephson plasmons (*57*), and charge-density wave amplitude modes (*58*), our observation represents a rare example where parametric amplification appears at the zone center with two distinct modes involved. Therefore, the experimental methodology and the concept of stimulated parametric amplification are applicable to a diverse range of quantum materials, providing insight into nonlinear mode-mode interactions that are often overlooked in equilibrium states.

## MATERIALS AND METHODS

### Synthesis of ErFeO_3_ single crystal

A single crystal of ErFeO_3_ (1.5 mm thick) grown by a floating zone melting technique was used in this work. The crystal was cut perpendicular to the *b* axis. The crystallographic axes were determined by Laue diffraction measurements. The detailed synthesis procedure and characterization has been reported previously (*15*).

### Time-domain THz spectroscopy and 2D THz spectroscopy

The majority of the output of a 1 kHz Ti:Sapphire laser amplifier (Coherent Legend Elite Duo, 800 nm, 12 mJ, 35 fs) was split into two identical arms. These beams were then recombined with a controlled relative time delay to generate a pair of single-cycle THz pulses via tilted pulse front optical rectification in Mg:LiNbO_3_ (*59*). The THz beams were then directed onto a sample using two off-axis parabolic mirrors arranged in a 4f configuration. The transmitted THz light was then recollimated and refocused by another pair of 4f parabolic mirrors onto a 2-mm thick ZnTe detection crystal where it was overlapped with the electro-optic sampling probe beam derived from a small portion of the fundamental laser power. A single-shot detection method was used to improve the signal-to-noise ratio by permitting the full time-dependence of the THz signal within a 20-ps window to be measured on a shot-to-shot basis (*34*, *60*). In time-domain THz spectroscopy experiments, one of the THz pulses was blocked while the remaining THz pulse was attenuated by a pair of WGPs to measure the primary linear response. For 2D THz measurements, differential chopping of the two pump beams (A and B) was used to extract the nonlinear signal as the interpulse time τ is scanned across the desirable range (*26*, *34*). The detected THz signals were kept within the linear response regime of the detection crystal, as verified by attenuating the signals and seeing a linear dependence of the measured signal on the field strength incident on the EO crystal and no change in the 2D spectrum; thus, the nonlinear signal originated solely from the response of the sample. Similar verification was conducted for our earlier measurements (*15*, *16*) as well. The nonlinear signal $H_{\text{NL}}$ is extracted as

$$H_{\text{NL}}\left(\tau ,t\right)=H_{\text{AB}}\left(\tau ,t\right)-H_{\text{A}}\left(\tau ,t\right)-H_{\text{B}}\left(\tau ,t\right)+H_{0}\left(\tau ,t\right)$$

where $H_{\text{AB}}$, $H_{\text{A}}$, $H_{\text{B}}$, and $H_{0}$ are the THz signals recorded with pump A, pump B, both pumps and no pumps, respectively. A 2D Fourier transform of the resulting time-domain signal yields the 2D THz spectrum.

## Supplementary Materials

### This PDF file includes:

Notes S1 to S5 Figs. S1 to S4 Table S1
