# Supplementary material for: Terahertz stimulated parametric downconversion of a magnon mode in an antiferromagnet
Source: Sci Adv. 2025 May 21;11(21):eadv3757. doi: 10.1126/sciadv.adv3757 (PMC12094239; doi:10.1126/sciadv.adv3757)
Supplement: Supplementary file 1 — Notes S1 to S5 Figs. S1 to S4 Table S1 [file sciadv.adv3757_sm.pdf]

Science Advances
AAAS

Supplementary Materials for

Terahertz stimulated parametric downconversion of a magnon mode in an antiferromagnet

Zhuquan Zhang *et al.*

Corresponding author: Keith A. Nelson, kanelson@mit.edu; Edoardo Baldini, edoardo.baldini@austin.utexas.edu; Shixun Cao, sxcao@shu.edu.cn; Zhuquan Zhang, zhuquan@mit.edu

Sci. Adv. **11**, eadv3757 (2025)
DOI: 10.1126/sciadv.adv3757

This PDF file includes:

Notes S1 to S5
Figs. S1 to S4
Table S1

Supplementary Note 1: Characterization of THz pump pulses.

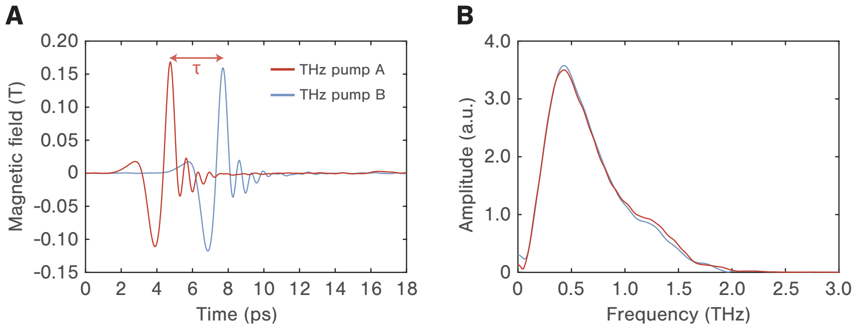

Fig. S1: Characterization of THz pump pulses. (**A**) Waveforms of THz pump pulses A and B used in all 2D THz spectroscopy experiments, with the variable inter-pulse delay set at $\tau = 3$ ps. During the measurements, the second THz pulse B is kept stationary, while τ is swept from 3 ps to 20 ps by varying the delay of the first THz pulse A. (**B**) Fourier transforms of both THz waveforms in (**A**) showing nearly identical spectral distributions.

Supplementary Note 2: Peak assignments of nonlinear responses.

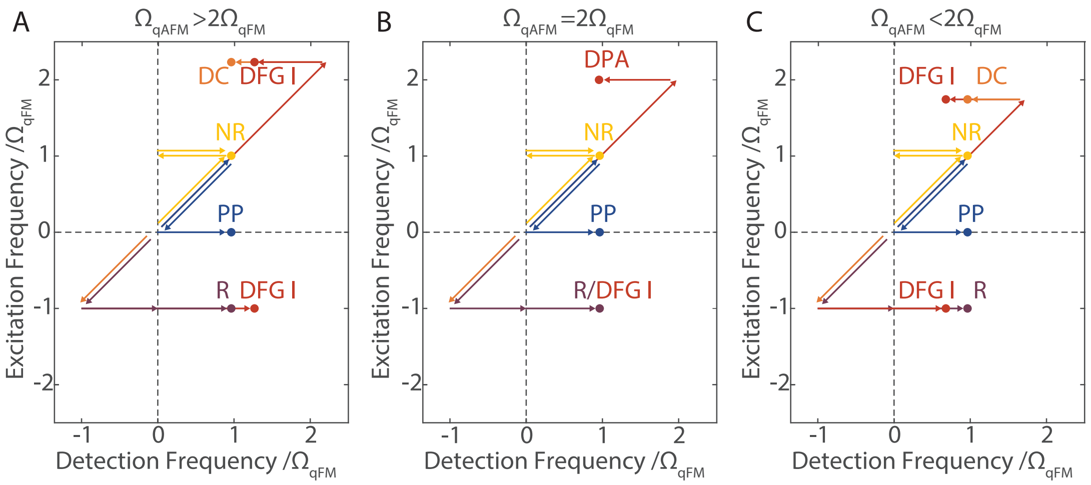

Fig. S2: Frequency vector representation. Assignment of all observed nonlinear responses in the 2D THz spectrum for three cases: $\Omega_{qAFM} > 2\Omega_{qFM}$ (**A**), $\Omega_{qAFM} = 2\Omega_{qFM}$ (**B**), and $\Omega_{qAFM} < 2\Omega_{qFM}$ (**C**).

In this section, we adopt a frequency-vector representation *(26, 36)* to assign the positions of all observed nonlinear signals in the 2D THz spectrum. We define the frequency vectors for the two THz pulses, labeled A and B, as $\mathbf{\Omega}_A = [\Omega_0, \Omega_0]$ and $\mathbf{\Omega}_B = [\Omega_0, 0]$. Here, Ω_0 can be either Ω_{qFM}, Ω_{qAFM}, or other frequencies (e.g., $\Omega_{qAFM} - \Omega_{qFM}$) if the THz field does not direcly excite any magnon m ode. Each field interaction from pulse A or B is represented by an addition of the corresponding frequency vector $\mathbf{\Omega}_A$ or $\mathbf{\Omega}_B$. Conjugate THz field interactions are represented by reversed vectors with opposite signs. For the third-order responses, the pump-probe (PP) response of the qFM mode is given by $\mathbf{\Omega}_{PP} = \mathbf{\Omega}_A - \mathbf{\Omega}_A + \mathbf{\Omega}_B = [\Omega_{qFM}, 0]$. In this case, the first THz pulse A interacts with the system twice, promoting it to a population state followed by a third field-

interaction with the second THz pulse B. The rephasing (R) and the non-rephasing (NR) signals are located at $\boldsymbol{\Omega}_R = -\boldsymbol{\Omega}_A + \boldsymbol{\Omega}_B + \boldsymbol{\Omega}_B = [\Omega_{qFM}, -\Omega_{qFM}]$ and $\boldsymbol{\Omega}_{NR} = \boldsymbol{\Omega}_A - \boldsymbol{\Omega}_B + \boldsymbol{\Omega}_B = [\Omega_{qFM}, \Omega_{qFM}]$, respectively, corresponding to the third-order responses in which only the first field interaction comes from THz pulse A and the other two field interactions are from THz pulse B. For the second-order responses, the field-driven downconversion (DC) signal arises if the qAFM mode is excited first, followed by a field interaction at frequency $\Omega_{qAFM} - \Omega_{qFM}$, which corresponds to $\boldsymbol{\Omega}_{DC} = [\Omega_{qAFM}, \Omega_{qAFM}] - [\Omega_{qAFM} - \Omega_{qFM}, 0] = [\Omega_{qFM}, \Omega_{qAFM}]$. By contrast, difference-frequency generation (DFG) involves the simultaneous excitation of both magnon modes, producing two contributions: $\boldsymbol{\Omega}_{DFG\ I} = -[\Omega_{qFM}, \Omega_{qFM}] + [\Omega_{qAFM}, 0] = [\Omega_{qAFM} - \Omega_{qFM}, -\Omega_{qFM}]$, and $\boldsymbol{\Omega}_{DFG\ II} = [\Omega_{qAFM}, \Omega_{qAFM}] - [\Omega_{qFM}, 0] = [\Omega_{qAFM} - \Omega_{qFM}, \Omega_{qAFM}]$. When the parametric resonance condition is satisfied ($\Omega_{qAFM} = 2\Omega_{qFM}$), the nonlinear interaction leads to $\boldsymbol{\Omega}_{DPA} = [\Omega_{qAFM}, \Omega_{qAFM}] - [\Omega_{qFM}, 0] = [\Omega_{qFM}, \Omega_{qAFM}]$ which overlaps with the field-driven downconversion signal. Figure S2 illustrates the relative positions of these peaks for $\Omega_{qAFM} > 2\Omega_{qFM}$, $\Omega_{qAFM} = 2\Omega_{qFM}$, and $\Omega_{qAFM} < 2\Omega_{qFM}$, corresponding to the scenarios at 150 K, 200 K, and 300 K in Fig. 3, respectively.

Supplementary Note 3: Detection of qAFM mode emission.

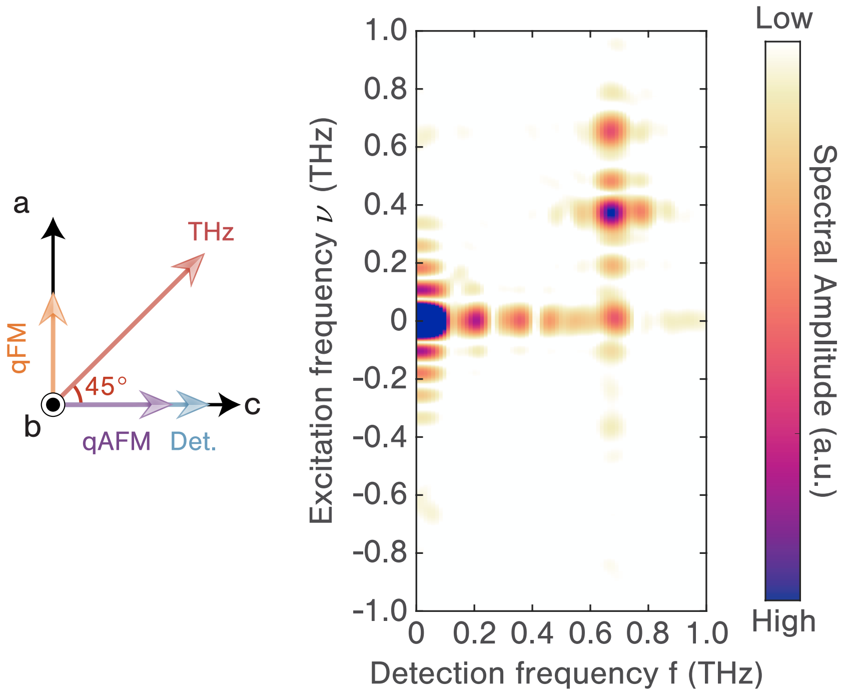

Fig. S3: 2D THz spectroscopy measurement with polarization selectivity of qAFM mode emission. For this measurement, the magnetic field components of both THz pulses are set at a 45° angle relative to both the a and c axes. The WGP is adjusted to selectively detect magnetization emissions along the c axis, while rejecting those along the a axis. The spectrum collected at room temperature only shows signals whose detection frequency corresponds to the frequency of the qAFM mode, i.e., $f = \Omega_{qAFM}$. The absence of DFG and downconversion signals confirms that the detected signals share the same polarization as the qFM mode emission.

Supplementary Note 4: Time-domain analysis of the stimulated parametric downconversion

In this section, we elaborate the time-domain signatures of the stimulated parametric downconversion of the magnon mode. Figure S4A shows the 2D time-domain traces of the nonlinear magnon signals $S(\tau, t)$ at 200 K, whose 2D Fourier transform corresponds to the 2D THz spectrum at 200 K presented in Fig. 3B. We conduct a 1D Fourier transform with respect to the inter-pulse delay τ, generating a 2D map that correlates the time-domain nonlinear signals with the excitation frequency ν. As depicted in Fig. S4B, within this 2D map, the majority of the nonlinear signals manifest with the excitation frequency either at Ω_{qAFM} or Ω_{qFM}, signifying their distinct origins. By extracting a slice along $\nu = \Omega_{qAFM}$, we can isolate the nonlinear signals initiated by the excitation of the qAFM mode, depicted in Fig. S4C. The time-domain waveform of these isolated signals exhibits an oscillatory response whose amplitude gradually increases over the initial few picoseconds, reaches a peak, and subsequently decays. This non-monotonic trend in the magnon response becomes more discernible through a short-time Fourier transform of the time-domain signals. The resulting time-dependent spectrogram, displayed in Fig. S4D, distinctly shows that the spectral weight of the signal consistently centers around 0.37 THz at all times, matching the frequency of the qFM mode (i.e., Ω_{qFM}). However, the maximum amplitude is observed around 6 ps. This delayed peak amplitude reflects the manifestation of magnon-magnon interaction, leading to gradual

buildup dynamics in the time domain. Unlike a field-driven downconversion process, which ceases immediately after the second THz pulse, the nonlinear magnon-magnon interaction persists as long as magnon coherence is maintained. Consequently, the amplitude of the qFM mode gradually increases before eventually decaying due to magnon decoherence. Such behavior suggests that although both the field-assisted downconversion and the degenerate parametric amplification processes are present at this temperature, the latter process dominates as the parametric resonance condition is satisfied (i.e., $\Omega_{qAFM} = 2\Omega_{qFM}$).

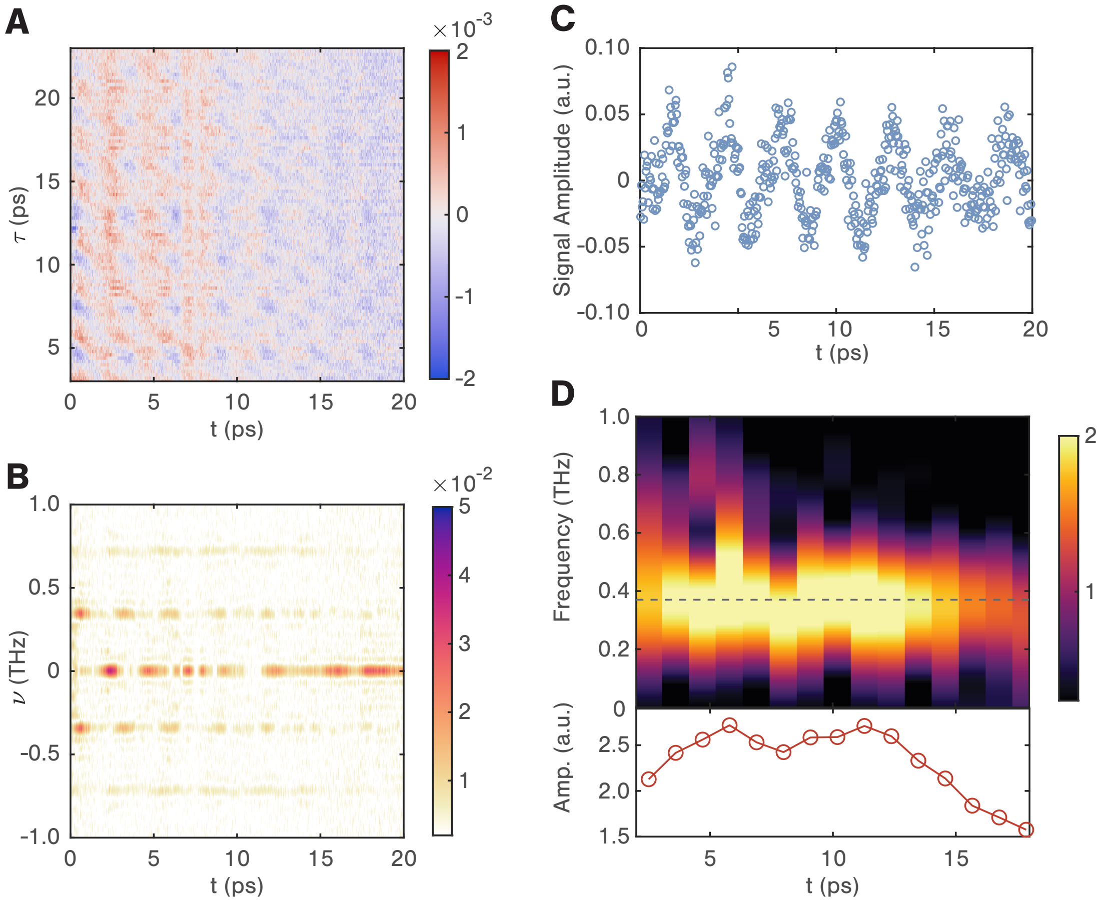

Fig. S4: Temporal evolution of magnon dynamics at 200 K. (**A**) Time-domain nonlinear THz responses plotted against inter-pulse delay τ and signal detection time t. (**B**) 1D Fourier transform of signals in (A) with respect to the inter-pulse delay, τ. (**C**) Temporal slice extracted from (B) at the excitation frequency $\nu = \Omega_{qAFM}$. (**D**) Top: Short-time Fourier transform of the time-domain signals in **C**. The dashed line indicates the frequency of the qFM mode, Ω_{qFM}, at 200 K. Bottom: Spectrogram slice at Ω_{qFM}. The signal amplitude gradually increases over the initial few picoseconds before exhibiting decay.

Supplementary Note 5: Theoretical analysis of nonlinear spin dynamics

A. Equations of motion

The interactions between the magnetic field components of the THz fields and the individual spins are governed by the Zeeman interaction. Consequently, the total Hamiltonian of the system can be expressed as a combination of the uniform two-spin Hamiltonian and the Zeeman interaction term, as follows:

$$\begin{aligned}\mathcal{H} &= \mathcal{H}_0 + \mathcal{H}_{Zeeman} \\ &= nJ\mathbf{S}_1 \cdot \mathbf{S}_2 + n\mathbf{D} \cdot (\mathbf{S}_1 \times \mathbf{S}_2) - \sum_{i=1,2} (K_a S_{ia}^2 + K_c S_{ic}^2) \\ &\quad - \gamma[\mathbf{H}_A(\tau, t) + \mathbf{H}_B(t)] \cdot (\mathbf{S}_1 + \mathbf{S}_2).\end{aligned} \tag{1}$$

To elucidate the two magnon modes in their eigenbasis, we perform a transformation by rotating the original crystallographic coordinates $\{a, b, c\}$ to form two new sets of coordinates $\{x_i, y_i, z_i\}$. This rotation aligns the equilibrium orientation of each sublattice spin $S_{i=1,2}$ along x_i ($S_{ix} = S, S_{iy} = S_{iz} = 0$) *(15, 27)*. Consequently, we obtain

$$\begin{bmatrix} S_{1x} \\ S_{1y} \\ S_{1z} \end{bmatrix} = \begin{bmatrix} -\cos\beta_0 & 0 & \sin\beta_0 \\ 0 & 1 & 0 \\ -\sin\beta_0 & 0 & -\cos\beta_0 \end{bmatrix} \begin{bmatrix} S_{1a} \\ S_{1b} \\ S_{1c} \end{bmatrix}, \begin{bmatrix} S_{2x} \\ S_{2y} \\ S_{2z} \end{bmatrix} = \begin{bmatrix} \cos\beta_0 & 0 & \sin\beta_0 \\ 0 & 1 & 0 \\ -\sin\beta_0 & 0 & \cos\beta_0 \end{bmatrix} \begin{bmatrix} S_{2a} \\ S_{2b} \\ S_{2c} \end{bmatrix},$$

where $\beta_0 = \frac{1}{2} \arctan \frac{nD}{nJ + K_a - K_c}$ is the canting angle between the $\mathbf{S}_2$ and the a axis.

In these newly defined coordinates, we introduce the following relations:

$$\sigma_x \equiv S_{2x} - S_{1x}, \qquad \sigma_y \equiv S_{2y} + S_{1y}, \qquad \sigma_z \equiv S_{2z} + S_{1z},$$

$$\gamma_x \equiv S_{2x} + S_{1x} - 2S, \qquad \gamma_y \equiv S_{2y} - S_{1y}, \qquad \gamma_z \equiv S_{2z} - S_{1z}.$$

Here, σ and γ represent the eigenmodes of the two-spin Hamiltonian in the absence of an external magnetic field. These correspond to the qFM and qAFM magnon modes, respectively. In the perturbative regime, which is relevant for our experiment, we assume that $S_{ix} \sim S \gg S_{iy},\ S_{iz}$. Therefore, the spin dynamics can be obtained by solving the equation of motion $\frac{d\mathbf{S}_i}{dt} = \frac{1}{i\hbar}[\mathbf{S}_i, \mathcal{H}]$. Without any external magnetic field, i.e., $\mathcal{H}_{Zeeman} = 0$, the leading order responses in S_{iy} and S_{iz} yield the following equations:

$$\begin{aligned}
\dot{\sigma_x} &= 0, \\
\dot{\sigma_y} &= \frac{2}{\hbar}(K_a - K_c)\cos(2\beta_0)S\sigma_z, \\
\dot{\sigma_z} &= -\frac{1}{\hbar}\left[n(J\cos(2\beta_0) + D\sin(2\beta_0) + J) + 2K_c\sin^2\beta_0 + 2K_a\cos^2\beta_0\right]S\sigma_y, \\
\dot{\gamma_x} &= 0, \\
\dot{\gamma_y} &= \frac{2}{\hbar}\left[n(J\cos(2\beta_0) + D\sin(2\beta_0)) + (K_a - K_c)\cos(2\beta_0)\right]S\gamma_z, \\
\dot{\gamma_z} &= -\frac{1}{\hbar}\left[n(J\cos(2\beta_0) + D\sin(2\beta_0) - J) + 2K_c\sin^2\beta_0 + 2K_X\cos^2\beta_0\right]S\gamma_y,
\end{aligned} \tag{2}$$

which describe the driven magnon modes in the linear response regime. The eigenfre-

quencies of the two modes can be solved as

$$\hbar\Omega_{\sigma,qFM} = 2S\left[n(J+K_a)(K_a-K_c)\right]^{\frac{1}{2}} \tag{3}$$

$$\hbar\Omega_{\gamma,qAFM} = S\left[4nJK_a + 4K_a(K_a-K_c) + n^2D^2\right]^{\frac{1}{2}}, \tag{4}$$

which are used to fit the experimental data in Fig. 2D of the main text. All parameter values used are presented in Table S1.

To clarify all second-order nonlinear responses sharing the same polarization as the qFM mode emission, we retain terms to second order in S_{iy} and S_{iz} and reformulate the equations in the magnon basis. This approach yields the following set of equations governing the nonlinear responses of the qFM mode:

$$\begin{aligned}
\dot{\sigma_x} =& \frac{1}{2\hbar}\{[n(J(\cos(2\beta_0)-1) + D\sin(2\beta_0)) + 2K_c\cos^2\beta_0 + 2K_a\sin^2\beta_0]\sigma_z\gamma_y \\
&+ [-n(J(\cos(2\beta_0)-1) + D\sin(2\beta_0)) + 2K_c\cos^2\beta_0 + 2K_a\sin^2\beta_0]\sigma_y\gamma_z\}, \\
&+ \gamma_0(\gamma_y\sin\beta_0 h_x - \sigma_y\cos\beta_0 h_z), \\
\dot{\sigma_y} =& \frac{1}{\hbar}\{2(K_a-K_c)\cos(2\beta_0)S\sigma_z + (K_a-K_c)\sin(2\beta_0)(2S\sigma_x - \sigma_z\gamma_z)\}, \\
&+ \gamma_0(-(2S\sin\beta_0 + \gamma_z\cos\beta_0)h_x - \sigma_z\sin\beta_0 h_z), \\
\dot{\sigma_z} =& \frac{1}{\hbar}\{-[n((J\cos(2\beta_0)+1) + D\sin(2\beta_0)) + 2K_c\sin^2\beta_0 + 2K_a\cos^2\beta_0]S\sigma_y \\
&+ (K_a-K_c)\sin(2\beta_0)\sigma_z\gamma_y\} + \gamma_0(\gamma_y\cos\beta_0 h_x + \sigma_y\sin\beta_0 h_z),
\end{aligned} \tag{5}$$

where h_k is the THz magnetic field component along the k coordinate. In these equations, terms proportional to $\gamma_j h_k$ are the sources responsible for the THz field-driven downconversion process. Specifically, they indicate that after the first THz field interaction excites the qAFM mode, a second THz field with a magnetic field component perpendicular to the net magnetization can initiate the downconversion process, thereby driving the qFM mode. On the other hand, terms proportional to $\sigma_j \gamma_k$ act as driving forces that are off-resonant with the qFM mode when the parametric resonance condition is not met, i.e., $\Omega_{qAFM} \neq 2\Omega_{qFM}$. These magnon-magnon mixing terms lead to nonlinear responses at difference and sum frequencies, sharing the same polarization as the qFM mode emission. However, when the parametric resonance condition is satisfied ($\Omega_{qAFM} = 2\Omega_{qFM}$), the difference frequency generation coincides with the qFM mode frequency, resulting in the resonant amplification of the qFM magnon mode.

B. Steady-state solutions of the second-order nonlinear responses

In this section, we analytically solve the steady-state responses of the two possible second-order terms that contribute to the stimulated magnon downconversion signal. We first consider the field-driven downconversion process. In this process, the qAFM mode is linearly excited by the THz magnetic field component along the c axis (i.e.,

$h_z = \mathbf{H}_z(\Omega_1)\exp(i\Omega_1 t)$), which leads to

$$\gamma_y(\Omega_1) = -\frac{2i\Omega_1}{\Omega_{qFM}^2 - \Omega_1^2 + i\Gamma\Omega_1}\gamma_0 S\cos\beta_0 \mathbf{H}_z(\Omega_1)\exp(i\Omega_1 t),$$
$$\gamma_z(\Omega_1) = -\frac{2}{\hbar}\cdot\frac{1}{\Omega_{qAFM}^2 - \Omega_1^2 + i\Gamma\Omega_1}\gamma_0 S^2[nJ(\cos(2\beta_0) - 1) + nD\sin(2\beta_0)$$
$$+ 2K_c\sin^2\beta_0 + 2K_a\cos^2\beta_0]\sin\beta_0\mathbf{H}_z(\Omega_1)\exp(i\Omega_1 t), \tag{6}$$

where Γ is a phenomenological damping constant. In the field-driven process ($\gamma \cdot h$), it is required that a magnetic field component of the second THz pulse align with the a axis, i.e., $h_x = \mathbf{H}_x(\Omega_2)\exp(i\Omega_2 t)$. Consequently, the equation of motion for the field-assisted downconversion process is determined as:

$$\ddot{\sigma}_z = -\omega_F^2\sigma_z + \gamma_0\cos\beta_0[\frac{S}{\hbar}(nJ(\cos(2\beta_0) + 1) + nD\sin(2\beta_0)$$
$$- 2K_c\sin^2\beta_0 - 2K_a\cos^2\beta_0)\mathbf{H}_x(\Omega_2)\gamma_z + \dot{\mathbf{H}}_x(\Omega_2)\gamma_y + \mathbf{H}_x(\Omega_2)\dot{\gamma_y}] \tag{7}$$

By setting $\Omega_1 = \Omega_{qFM}$ and $\Omega_2 = \Omega_{qAFM} - \Omega_{qFM}$, the steady-state response is given by

$$\sigma_{z,\gamma h}(\Omega_{qFM}) = 2S\gamma_0^2\cos^2\beta_0\sin\beta_0\mathbf{H}_x(\Omega_{qAFM} - \Omega_{qFM})\mathbf{H}_z(\Omega_{qAFM})$$
$$\cdot\frac{e^{i\Omega_{qFM}t}}{\hbar\Gamma\Omega_{qFM}\Omega_{qAFM}}[S^2(n^2(J\cos 2\beta_0 + D\sin 2\beta_0)^2 -$$
$$(nJ - 2K_c\sin^2\beta_0 - 2K_a\cos^2\beta_0)^2) - \hbar^2\Omega_{qFM}\Omega_{qAFM}]. \tag{8}$$

Similarly, for the degenerate parametric amplification process, the first THz field drives the qAFM mode (γ), while the second THz field directly excites the qFM mode (σ).

The linear responses of the qFM mode are derived as:

$$\begin{aligned}
\sigma_y(\Omega_2) =& \frac{2}{\hbar} \cdot \frac{i\Omega_2}{\Omega_{qFM}^2 - \Omega_2^2 + i\Gamma\Omega_2} \gamma_0 S \sin\beta_0 \mathbf{H}(\Omega_2) \exp(i\Omega_2 t), \\
\sigma_z(\Omega_2) =& - \frac{2}{\hbar^2} \cdot \frac{1}{\Omega_{qFM}^2 - \Omega_1^2 + i\Gamma\Omega_2} \gamma_0 S^2 [n((J\cos(2\beta_0) + 1) + D\sin(2\beta_0)) \\
& + 2K_c \sin^2\beta_0 + 2K_a \cos^2\beta_0] \sin\beta_0 \mathbf{H}(\Omega_2) \exp(i\Omega_2 t).
\end{aligned} \tag{9}$$

In equation (6), the magnon-magnon mixing terms involving σ_x result in the emission of a magnetic field at a difference frequency. The resulting transient magnetization is given by $h_x \sim \mu\frac{N}{V}\hbar\gamma_0\sigma_x$, where μ represents the magnetic susceptibility, and $\frac{N}{V}$ denotes the spin density of the system.Consequently, the nonlinear equation of motion that delineates the degenerate parametric amplification process is given by:

$$\begin{aligned}
\ddot{\sigma_y} =& - \Omega_{qFM}^2 \sigma_y - \gamma_0^2 S \sin\beta_0 \frac{\mu N}{V} \\
& [(nJ(\cos 2\beta_0 - 1) + nD\sin 2\beta_0 + 2K_c\cos^2\beta_0 + 2K_a \sin^2\beta_0)\sigma_z\gamma_y \\
& - (nJ(\cos 2\beta_0 - 1) + nD\sin 2\beta_0 - 2K_c\cos^2\beta_0 - 2K_a \sin^2\beta_0)\sigma_y\gamma_z] \\
=& - \Omega_{qFM}^2 \sigma_y - \frac{\gamma_0^2 S^2}{\hbar} \sin\beta_0 \frac{\mu N}{V} [\frac{1}{i\Omega_{qFM}} n^2((J\cos 2\beta_0 + D\sin 2\beta_0)^2 - J^2) \\
& - \frac{1}{i\Omega_{qAFM}} n^2 (J(\cos 2\beta_0 - 1) + D\sin 2\beta_0)^2]\sigma_y\gamma_y,
\end{aligned} \tag{10}$$

where, in the final step, σ_x and γ_x are transformed to σ_y and γ_y, respectively, in accordance with their linear responses as derived in equation (9). By solving for the

steady-state response of the degenerate parametric amplification, we obtain:

$$\sigma_{z,\gamma\sigma}(\Omega_{qFM}) = \frac{4\gamma_0^4 S^5}{\hbar^2}\frac{1}{i\Omega_{qFM}\Gamma}\cos\beta_0\sin^2\beta_0 \cdot \frac{\mu N}{V} n(J(\cos 2\beta_0 + 1) + D\sin 2\beta_0)$$
$$[\frac{1}{i\Omega_{qFM}} n^2((J\cos 2\beta_0 + D\sin 2\beta_0)^2 - J^2) - \frac{1}{i\Omega_{qAFM}} n^2(J(\cos 2\beta_0 - 1) + D\sin 2\beta_0)^2]$$
$$\cdot \int d\Omega_{qFM} \frac{\Omega(\Omega_{qFM} - \Omega)}{\Omega_{qAFM}^2 - \Omega^2 + i\Gamma\Omega} \cdot \frac{\mathbf{H}_x(\Omega_{qFM} - \Omega)\mathbf{H}_z(\Omega)}{\Omega_{qFM}^2 - (\Omega - \Omega_{qFM})^2 - i\Gamma(\Omega - \Omega_{qFM})}. \quad (11)$$

Here, we model the frequency-dependent THz pump spectrum $\mathbf{H}(\Omega)$ as:

$$\mathbf{H}(\Omega) = \mathbf{H}_0 \frac{\Omega^2}{\Omega_0^2} exp(-\frac{\Omega^2}{\Omega_0^2}), \quad (12)$$

where $\Omega_0 = 0.5$ THz. This function closely matches the measured THz spectral distribution shown in Fig. S1 and is used as the input for solving the two steady-state responses (equations 8 and 11). The resulting solutions and their sum are plotted in Fig. 4C of the main text.

Parameter	Value
Total spin S	$5/2$
Nearest neighboring number n	6
Exchange constant J	4.6 (meV)
Antisymmetric exchange constant D	0.1066 (meV)
Temperature-independent magnetic anisotropy K_a	0.00863 (meV)
Temperature-dependent magnetic anisotropy K_c	$\frac{0.9847}{T-27.62} + 0.00423$ (meV)

Table S1:Parameter values used for fitting the magnon frequencies. Realistic values from the literature are used here *(29, 31, 32)*. The temperature-dependent magnetic anisotropy K_c is obtained by fitting the experimentally observed magnon frequencies to a modified Curie–Weiss law. *(31)*
